# Supplementary material for: Topological band structure via twisted photons in a degenerate cavity
Source: Nat Commun. 2022 Apr 19;13:2040. doi: 10.1038/s41467-022-29779-3 (PMC9018724; doi:10.1038/s41467-022-29779-3)
Supplement: Supplementary file 1 — Supplementary Information [file 41467_2022_29779_MOESM1_ESM.pdf]

Supplementary Information for: Topological band structure via twisted photons in a degenerate cavity

Mu Yang[a], Hao-Qing Zhang[a], Yu-Wei Liao[a], Zheng-Hao Liu, Zheng-Wei Zhou, Xing-Xiang Zhou, Jin-Shi Xu,[†] Yong-Jian Han,[‡] Chuan-Feng Li,[§] and Guang-Can Guo

CAS Key Laboratory of Quantum Information,

University of Science and Technology of China,

Hefei 230026, People's Republic of China and

CAS Center For Excellence in Quantum Information and Quantum Physics,

University of Science and Technology of China, Hefei 230026, People's Republic of China

[a] These authors contribute equally to this work

CONTENTS

I. Details of the experimental setup 2

II. Dispersion relation of the cavity 4

III. Direct measurement of the density of states 6

IV. The transmission modes of the cavity 8

V. Direct measurement of the energy band spectrum 10

VI. Photon distributions after the modulation via SLM 10

VII. Direct measurement of the topological winding 10

VIII. Edge effect and disorder effect 11

References 11

I. DETAILS OF THE EXPERIMENTAL SETUP

We use the device shown in FIG. S1 to investigate the topological properties of the spin-orbit coupling system. We use an infrared continuous wave (CW) laser with the Gaussian mode at $\lambda = 880$ nm. The polarization of the laser is prepared to be left or right circular ($\circlearrowleft$ or $\circlearrowright$) after passing through the polarization beam splitter (PBS) and a quarter-wave plate (QWP) with the optical-axis setting at 45°. The photons are coupled into the cavity by the first mirror with a ratio between transmission and reflection of 5/95. The photons that are not coupled into the cavity are detected by a photodetector (PD). Since the constructed degenerate optical resonant cavity supports all the Laguerre-Gauss (LG) modes that form a complete basis, the input laser mode does not need to be specially adjusted.

The degenerate optical cavity has been theoretically investigated [1, 2]. In experiment, the degenerate cavity consist of two plane mirrors and two lenses of focal length $f = 0.1$ m. The free spectral range (FSR) of the cavity is about 375 MHz, while the linewidth is about 13.6 MHz. A Q-plate with $q = 1$ is placed in the center of the cavity, on which the electrostatic field is controlled by an arbitrary function generator (AFG). A (η/π)-wave plate (WP) is set behind to rotate the polarization (e.g., $\eta = \pi/4$ for the quarter-wave plate). To scan the cavity length ΔL, a piezoelectric transducer (PZT) is pasted on the second mirror and is driven by an amplified periodic triangular wave signal generated by the AFG. The scanning frequency

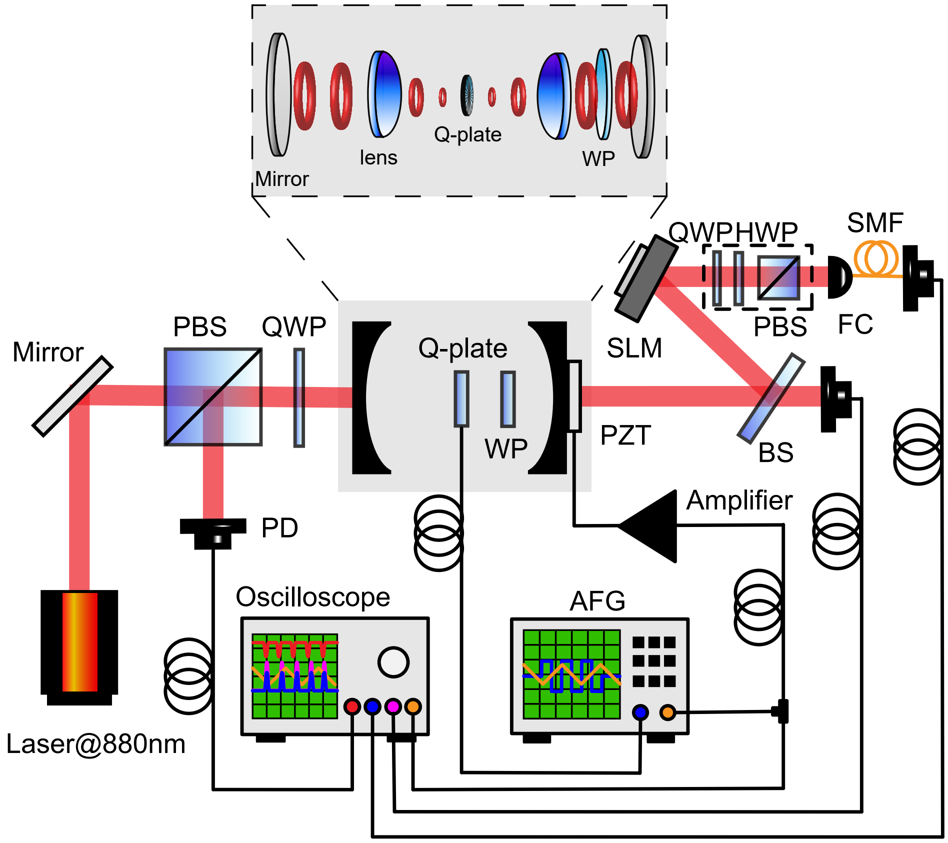

Figure S1. **Experimental setup.** The cavity of 0.4 m length is pumped by an infrared laser beam at 880 nm in a Gaussian mode. A quarter-wave plate (QWP) is used to rotate the polarization of the incident beam. A photodetector (PD) in the reflection path of the polarization beam splitter (PBS) is used to detect the reflected optical intensity from the cavity. The transmitted signal is divided into two paths by a beam splitter (BS). One of which is detected directly and the other is modulated firstly by a spatial light modulator (SLM) and then coupled to a single-mode fiber (SMF). A QWP, a half-wave plate (HWP), and a PBS in the dotted box before the fiber coupler (FC) is used for the post-selection of polarization. An arbitrary function generator (AFG) modulates the Q-plate and periodically drives the Piezoelectric Transducer (PZT) attached to the output couple BS of the cavity after amplification to change the length of the cavity. All electrical signals are recorded by the oscilloscope. The inset shows the details of the cavity which consists of two Mirrors and two lenses. The Q-plate and a wave-plate (WP) in the cavity are used to modulate the photon modes, which are represented as red circles.

of the triangular wave signal is set at 40 Hz such that the system reaches a steady state at each frequency. To reduce the cavity's dissipation (α), all the optical elements in the cavity are coated with anti-reflection films. The linewidth of the cavity can be reduced to obtain sharper transmission peaks.

The photons are out-coupled by the second mirror with the ratio of 1/99. The output photons are separated into two paths by a beam splitter (BS). The transmitted photons are detected directly by a PD, while

the reflected parts are first modulated by a spatial light modulator (SLM) and post-selected by a single mode fiber (SMF). When detecting topological windings, a QWP, a half-wave plate (HWP), and a PBS in the dashed panel are set before the fiber coupler (FC) for the post-selection of polarization. All the signals detected by the PDs are recorded in an oscilloscope with a 1 GHz bandwidth, which allows readind the system's eigenenergy directly.

In our experiment, the length of the synthetic dimension is limited by the size of the minimum aperture of the Q-plate. With the increasing of topological charge number m of orbital angular momentum (OAM) modes, the transverse radius of maximum field amplitude r_m will increase as $r_m = \omega_0\sqrt{m/2}$ [3]. ω_0 represents the waist radius of Gaussian mode ($m = 0$). In this experiment, the radius of the Q-plate (minimal aperture) is 0.25 mm and ω_0 is about 80 μm. The maximal topological charge number m is $2r_m^2/\omega_0^2 \approx 1.95 \times 10^3$. The cavity supports the even order OAM modes ranging $-1.95 \times 10^3 < m < 1.95 \times 10^3$ and the length of the synthetic dimension is about 1.95×10^3.

On the other hand, the cavity loss determines the average lifetime of photons. The total loss α of the cavity is about 0.1. The average lifetime of photons becomes $\tau = L/(\alpha c) = 10L/c$ with L and c representing the length of the cavity and the speed of the photons, respectively. Therefore, the photons can pass through the Q-plate 10 times on average. The average charges of degenerate orbital angular momenta inside the degenerate cavity is located in $m \in [-20, 20]$. The lifetime can significantly increase by introducing an optical amplifier into the cavity.

II. DISPERSION RELATION OF THE CAVITY

In this section, we derive the dispersion relation for the cavity without input and output. As the photons propagate in a steady cavity, the state $|\phi(t)\rangle = (..., \phi(t)_{\circlearrowleft,m-1}, \phi(t)_{\circlearrowright,m-1}, \phi(t)_{\circlearrowleft,m}, \phi(t)_{\circlearrowright,m}, ...)$ at time t must satisfy the condition of mode self-reproduction, denoted as

$$|\phi(t+T)\rangle = e^{-i\beta L}\,|\phi(t)\rangle\,, \tag{S1}$$

where $\beta = \Omega/c + i\alpha$ and L is one round trip (one period) length of the cavity. Ω is the resonant frequency of the vacuum cavity and α is the attenuation coefficient. $T = L/c$, where c represnts the speed of light. On the other hand, according from the method, the action of Q-plate ($q = 1$) is described as

$$J_{Q(\delta)} = \sum_m \cos(\delta/2)(a^\dagger_{\circlearrowright,m}a_{\circlearrowleft,m} + a^\dagger_{\circlearrowleft,m}a_{\circlearrowright,m}) + i\sin(\delta/2)(a^\dagger_{\circlearrowright,m+2q}a_{\circlearrowleft,m} + \text{h.c.}). \tag{S2}$$

The action of η/π-wave plate (WP) can be described as

$$J_{\lambda(\eta)} = \sum_m \cos(\eta)(a^\dagger_{\circlearrowright,m}a_{\circlearrowleft,m} + a^\dagger_{\circlearrowleft,m}a_{\circlearrowright,m}) + i\sin(\eta)(a^\dagger_{\circlearrowright,m}a_{\circlearrowleft,m} + \text{h.c.}). \tag{S3}$$

When photons pass a round trip through the Q-plate and η/π-wave plate, the operation on the photons in the cavity can be described as

$$\hat{U} = J_{Q(\delta)} J_{\lambda(\eta)} J_{\lambda(\eta)} J_{Q(\delta)}. \tag{S4}$$

When the evolutionary period is guaranteed, the photon state in the cavity satisfies

$$|\phi(t+T)\rangle = \hat{U} |\phi(t)\rangle . \tag{S5}$$

According to Eq. S1 and S5, we find

$$e^{-i\beta L} |\phi(t)\rangle = \hat{U} |\phi(t)\rangle . \tag{S6}$$

Here we define an effective Hamiltonian of $\hat{H}_{\text{eff}} = i \log \hat{U}$. In the quasi-momentum space, we define the Bloch modes $|k\rangle = \sum_j e^{-ijk} |j\rangle$ $(j = m/2)$. The Hamiltonian is denoted as $\hat{H}_{\text{eff}}(k) = \mathbf{n}(k) \cdot \boldsymbol{\sigma} E_k |k\rangle \langle k|$, where $\mathbf{n}(k) = [n_x(k), n_y(k), n_z(k)]$ is an unit vector, and $\boldsymbol{\sigma} = [\sigma_x, \sigma_y, \sigma_z]$ is the Pauli matrix. The eigenstate of $\hat{H}_{\text{eff}}(k)$ can be written as $|\phi_k^s\rangle = |\psi_k^s\rangle |k\rangle$, where $|\psi_k^s\rangle = (\psi^s_{\circlearrowleft,k}, \psi^s_{\circlearrowright,k})^T$ and Eq. S6 becomes

$$e^{-i\beta L} |\psi_k^s\rangle = \hat{U}_k |\psi_k^s\rangle , \tag{S7}$$

where $s = \pm 1$ related to the SAM denotes the upper and lower energy bands. The unitary evolution in the momentum space becomes $\hat{U}_k = J_{Q_k(\delta)} J_{\lambda_k(\eta)} J_{\lambda_k(\eta)} J_{Q_k(\delta)}$. $J_{Q_k(\delta)}$ and $J_{\lambda_k(\eta)}$ represent the operations of Q-plate and WP in reciprocal space, respectively, which are given by

$$J_{Q_k(\delta)} = \begin{bmatrix} \cos(\delta/2) & i \sin(\delta/2) e^{-ik} \\ i \sin(\delta/2) e^{ik} & \cos(\delta/2) \end{bmatrix}, \tag{S8}$$

and

$$J_{\lambda_k(\eta)} = \begin{bmatrix} \cos(\eta) & i \sin(\eta) \\ i \sin(\eta) & \cos(\eta) \end{bmatrix}. \tag{S9}$$

The Eq. S7 can be rewritten as

$$e^{-i\beta L} |\psi_k^s\rangle = \hat{U}_k |\psi_k^s\rangle = e^{-i\hat{H}_{\text{eff}}(k)} |\psi_k^s\rangle . \tag{S10}$$

The evolution satisfies $\hat{U}_k = \cos(E_k) I + i \sin(E_k) \mathbf{n}(k) \cdot \boldsymbol{\sigma}$. Compared with the Eq. S10, we can obtain

$$\begin{gathered} sE_k(\eta, \delta) = s \cos^{-1}[\sin 2\eta \cos k \sin \delta - \cos 2\eta \cos \delta], \\ sn_x = [\cos 2k \sin^2 \frac{\delta}{2} \sin 2\eta - \cos k \cos 2\eta \sin \delta - \cos^2 \frac{\delta}{2} \sin 2\eta] / \sin sE_k, \\ sn_y = [\sin 2k \sin^2 \frac{\delta}{2} \sin 2\eta - \sin k \cos 2\eta \sin \delta] / \sin sE_k, \\ sn_z = 0, \end{gathered} \tag{S11}$$

where E_k is the energy dispersion relation of the cavity. The unit vector $\mathbf{n}(k) = [n_x(k), n_y(k), n_z(k)]$ reveals the topological winding numbers of the system as discussed later. We can observe the windings of the unit vector $\mathbf{n}(k)$ and $-\mathbf{n}(k)$ for the upper and lower bands. Moreover, we can find $\hat{H}_{\text{eff}}(k)$ meets $\Gamma\hat{H}_{\text{eff}}(k)\Gamma = -\hat{H}_{\text{eff}}(k)$ with $\Gamma = \sigma_z$, which means the system has chiral symmetry.

The timeframes are the time evolution with different starting points, which are unique properties in the periodically driven system. If the photons pass a round through η/π-WP firstly and then the Q-plate, denoted as the second time frame, the evolution operator $\hat{U}_k'$ can be rewritten as

$$\hat{U}_k' = J_{\lambda_k(\eta)} J_{Q_k(\delta)} J_{Q_k(\delta)} J_{\lambda_k(\eta)}. \tag{S12}$$

Similarly, the energy dispersion and unit victors of the second timeframe are given by

$$\begin{aligned} sE_k'(\eta,\delta) &= s\cos^{-1}[\sin 2\eta \cos k \sin\delta - \cos 2\eta\cos\delta], \\ sn_x' &= -[\cos k\cos 2\eta\sin\delta + \cos\delta\sin 2\eta]/\sin sE_k, \\ sn_y' &= -[\sin k\sin\delta]/\sin sE_k, \\ sn_z' &= 0. \end{aligned} \tag{S13}$$

Obviously, these two time frames have the same energy dispersion relation but the different three-dimensional unit vector $\mathbf{n}(k)$. The winding number of the unit vector $n(k)$ is the topological invariant, protected by the chiral symmetry. Therefore, the two timeframes have different topological invariants and correspond to different topologies.

III. DIRECT MEASUREMENT OF THE DENSITY OF STATES

In this section, we turn to an open system and demonstrate the method to directly measure the density of states (DOS) from the cavity output. The coupling of the cavity mirror can be described by [4, 5]

$$\begin{bmatrix} \phi_{out} \\ a \end{bmatrix}_{\circlearrowleft(\circlearrowright),m} = \begin{bmatrix} r & \kappa \\ -\kappa^* & r^* \end{bmatrix} \begin{bmatrix} \phi_{in} \\ b \end{bmatrix}_{\circlearrowleft(\circlearrowright),m}, \tag{S14}$$

where $\kappa = i|\kappa|$ and $r = |r|$. m represents the OAM topological charge. ϕ_{in} (ϕ_{out}) represents the input (output) photonic state and a (b) represents the state before (after) modulation in the cavity. Consider phase accumulations as the photons propagate around the cavity, the photonic amplitude $a_{\circlearrowleft(\circlearrowright),m}$ should be rewritten as

$$a_{\circlearrowleft(\circlearrowright),m} \rightarrow e^{-i\beta L} a_{\circlearrowleft(\circlearrowright),m}. \tag{S15}$$

Combining Eq. S14 and S15, we find

$$b_{\circlearrowleft(\circlearrowright),m} = \frac{1}{r^*}(e^{-i\beta L} a_{\circlearrowleft(\circlearrowright),m} + \kappa^* \phi_{in,\circlearrowleft(\circlearrowright),m}), \tag{S16}$$

and

$$\phi_{out,\circlearrowleft(\circlearrowright),m} = \frac{1}{r^*}(\kappa e^{-i\beta L} a_{\circlearrowleft(\circlearrowright),m} + \phi_{in,\circlearrowleft(\circlearrowright),m}). \tag{S17}$$

Representing the states as the state vectors, Eq. S16 and Eq. S17 become

$$|b\rangle = \frac{1}{r^*}(e^{-i\beta L}|a\rangle + \kappa^*|\phi_{in}\rangle), \tag{S18}$$

and

$$|\phi_{out}\rangle = \frac{1}{r^*}(\kappa e^{-i\beta L}|a\rangle + |\phi_{in}\rangle), \tag{S19}$$

where $|\phi_{in}\rangle = (..., \phi_{in,\circlearrowleft,m-1}, \phi_{in,\circlearrowright,m-1}, \phi_{in,\circlearrowleft,m}, \phi_{in,\circlearrowright,m}, ...)$, so are $|a\rangle$, $|b\rangle$ and $|\phi_{out}\rangle$.

By taking $|b\rangle = \hat{U}|a\rangle$ into Eq. S18, we can get

$$\frac{1}{r^*}(e^{-i\beta L}|a\rangle_n + \kappa^*|\phi_{in}\rangle) = \hat{U}|a\rangle_{n-1}, \tag{S20}$$

where n represents the loop number of the photons running in the cavity. Note that $|a\rangle_{n-1} = |a\rangle_n$ if $n \to \infty$. Initially, there is no photon in the cavity, which means $|a\rangle_0 = \mathbf{0}$. After n loop number, we get

$$|a\rangle_n = -\kappa^* e^{i\beta L}\sum_n (r^*)^n e^{in\beta L}\hat{U}^n|\phi_{in}\rangle. \tag{S21}$$

Combining the Eq. S19 and S21, we can get the output state as

$$|\phi_{out}\rangle = \frac{1}{r^*}|\phi_{in}\rangle - \frac{|\kappa|^2}{r^*}\sum_n (r^*)^n e^{in\beta L}\hat{U}^n|\phi_{in}\rangle. \tag{S22}$$

The first term on the right-hand side represents the direct reflection of $|\phi_{in}\rangle$. The second term represents the transmission of the field, and we redefine the $|\phi_{out}\rangle$ as

$$|\phi_{out}\rangle = -\frac{|\kappa|^2}{r^*}\sum_n (r^*)^n e^{in\beta L}\hat{U}^n|\phi_{in}\rangle. \tag{S23}$$

The eigenstates $|\phi_k^s\rangle = |\psi_k^s\rangle|k\rangle$ of the Hamiltonian $\hat{H}_{\text{eff}}(k)$ form a set of complete basis for expanding $|\phi_{out}\rangle$. We set the input field to be $|\phi_{in}\rangle = |\phi_{in}^s\rangle|m_0\rangle$ with $|m_0\rangle$ representing a special momentum state. The transmission field $|\phi_{out}\rangle$ can be written as

$$\begin{aligned} |\phi_{out}\rangle &= -\tfrac{|\kappa|^2}{r^*}\textstyle\sum_{k,s}\sum_{n\to\infty}(r^*)^n e^{in\beta L}e^{-inE_k}\langle k|m_0\rangle\langle\psi_k^s|\phi_{in}^s\rangle|\psi_k^s\rangle|k\rangle \\ &= \textstyle\sum_{k,s}\frac{-|\kappa|^2/r^*}{1-r^*e^{-i(sE_k-\beta L)}}\langle k|m_0\rangle\langle\psi_k^s|\phi_{in}^s\rangle|\psi_k^s\rangle|k\rangle, \end{aligned} \tag{S24}$$

where sE_k represents the eigenenergy of the Hamiltonian $\hat{H}_{\text{eff}}$. Taking $\Delta L = L - 2n\pi/\beta$ ($n \in \mathbb{N}^+$ and $\beta\Delta L < 2\pi$), we define the transmission coefficient T_k^s as

$$T_k^s = \frac{-|\kappa|^2/r}{1 - re^{-i(sE_k-\beta\Delta L)}}\langle k|m_0\rangle\langle\psi_k^s|\phi_{in}^s\rangle. \tag{S25}$$

The intensity $I_o = |\phi_{out}|^2$ of the output field is

$$\begin{aligned} I_o &= \sum_s \sum_{kk'} \langle k^{'}| \langle \psi^s_{k'}| (T^s_{k'})^* T^s_k |\psi^s_k\rangle |k\rangle \\ &= \sum_s \sum_{kk'} \delta(k,k^{'}) (T^s_{k'})^* T^s_k \\ &= \sum_{k,s} |T^s_k|^2 \\ &= \sum_{k,s} \frac{|\kappa|^4/|r|^2}{1+|r|^2-2|r|\cos(sE_k-\beta\Delta L)} |\langle k|m_0\rangle | \langle \psi^s_k|\phi^s_{in}\rangle |^2. \end{aligned} \tag{S26}$$

By choosing an appropriate input state $|\phi^s_{in}\rangle$, $\sum_s |\langle k|m_0\rangle \langle \psi^s_k|\phi^s_{in}\rangle|^2$ could be independent on k. For instance, the input state in our experiment with the Gaussian mode $|m_0 = 0\rangle$ is prepared to the maximally mixed polarization state of $1/2(|\circlearrowleft\rangle\langle\circlearrowleft| + |\circlearrowright\rangle\langle\circlearrowright|)$, the total intensity I_o becomes

$$\begin{aligned} I_\text{o} &= \sum_k \frac{|\kappa|^4/|r|^2}{1+|r|^2-2|r|\cos(sE_k-\beta\Delta L)} |\langle k|0\rangle|^2 (|\langle \psi^s_k|\circlearrowleft\rangle|^2 + |\langle \psi^s_k|\circlearrowright\rangle|^2) \\ &= \sum_k \frac{|\kappa|^4/|r|^2}{1+|r|^2-2|r|\cos(sE_k-\beta\Delta L)}. \end{aligned} \tag{S27}$$

On the other hand, the density of states related to volume V is defined as

$$D(E) = \frac{1}{V}\sum_k \delta[E - E_k], \tag{S28}$$

where E_k represents the energy band along momentum k. In our experiment, only $sE_k = \beta\Delta L$ mainly contribute to the transmission intensity $I_\text{o}(\beta\Delta L)$ in Eq. S27. When $|r| \to 1$, $\frac{1}{1+|r|^2-2|r|x}$ will very close to the $\delta(x-1)$ function ($x = \cos(sE_k - \beta\Delta L)$). Thus, I_o can be approximated as

$$I_\text{o}(\beta\Delta L) \approx \Gamma \sum_k \delta(\beta\Delta L - sE_k), \tag{S29}$$

where Γ is the normalised coefficient. Regarding Γ as the volume V, I_o is denoted as density of the states

$$I_\text{o}(\beta\Delta L) = D(\beta\Delta L). \tag{S30}$$

IV. THE TRANSMISSION MODES OF THE CAVITY

Here we illustrate more details of the output modes of the cavity with $\eta = \pi/4$, which is shown in Fig. S2. The input photons are on the Gaussian mode ($m = 0$) with horizontal polarization. When $\delta = 0$, the transverse mode of light is always kept in the Gaussian mode ($m = 0$), but the polarization changes periodically. As a result, there are the splittings of the transmission peaks, which are twice that in the vacuum cavity. When $\delta > 0$, the high order angular momentum modes begin mixed, and the spectra of the system getting more and more complicated, which satisfies the dispersion relation described in Eq. S11. Especially when $\delta = \pi/2$, the transverse modes are restricted to the angular momentum modes

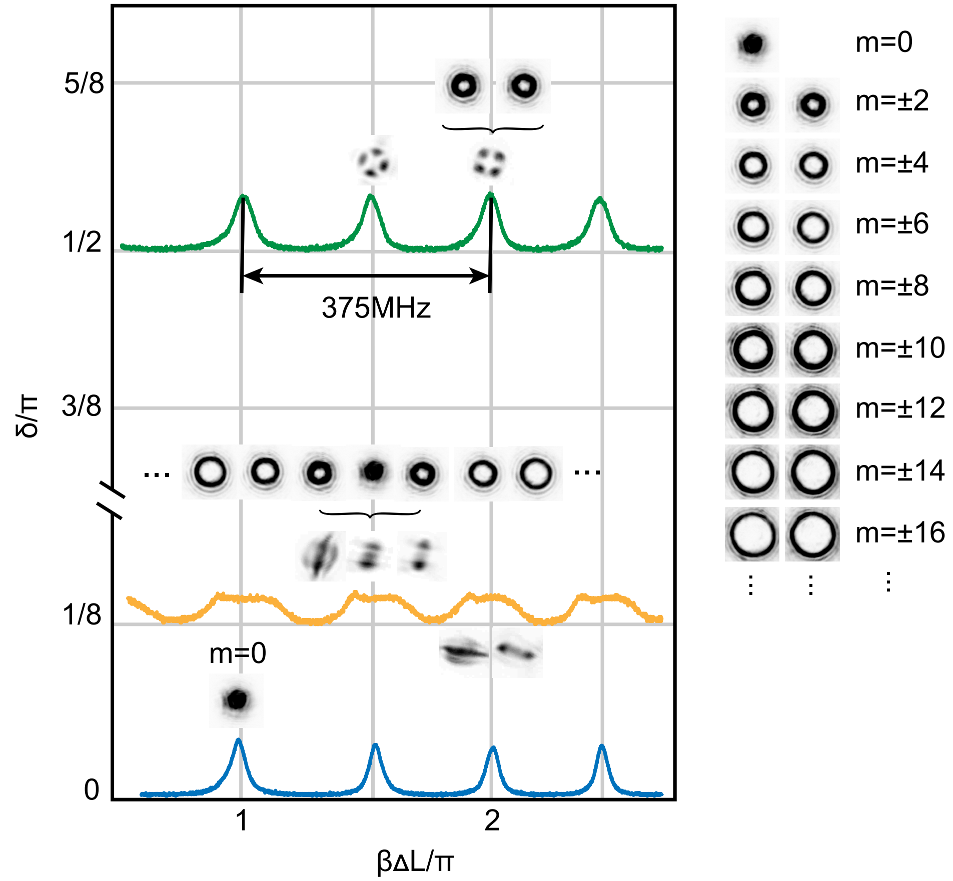

Figure S2. **Experimental transmission modes at** $\eta = \pi/4$**.** The spectra are labled in blue ($\delta = 0$), yellow ($\delta = 1/8$) and green ($\delta = \pi/2$). The intensity distributions of transmitted photons, shown near the spectra, reveal the mixing of different angular momentum modes. The experimental spatial distributions of different angular momentum modes ($|m| = 0 \sim 16$) are shown on the right panel.

with topological charge $|m| \leq 1$, which leads to the same spectrum of $\delta = 0$. The images near the spectra represent the transmission intensity distributions, which are detected by a high-speed camera. These intensity distributions are formed by mixing the simple OAM "ring" patterns. See the supplementary video for more details.

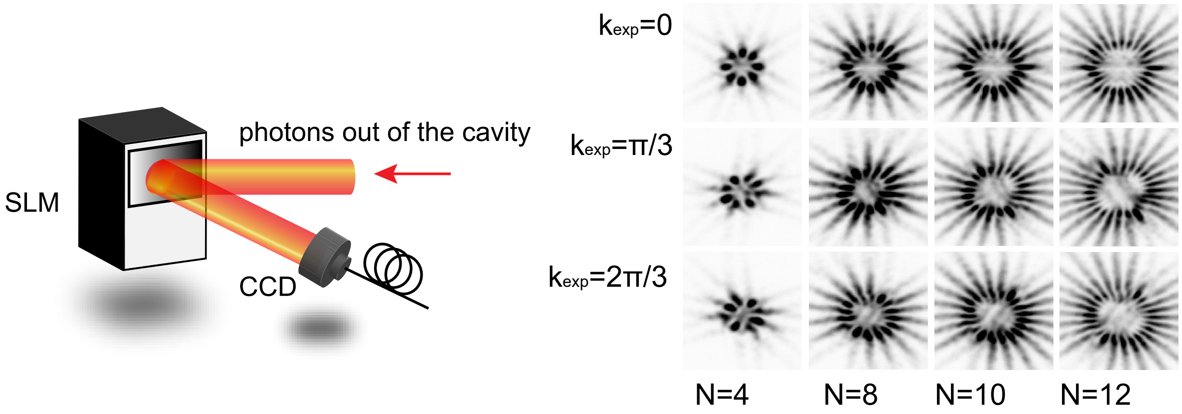

Figure S3. Experimental setup for post-selecting on the basis $|k_{\rm exp}\rangle\langle k_{\rm exp}|$. SLM: spatial light modulator; CCD: charge coupled device camera; (left). The photon distributions after post-selected by SLM with different settings of holograms (right).

V. DIRECT MEASUREMENT OF THE ENERGY BAND SPECTRUM

With the post-selected state $|k\rangle$, the transmission intensity becomes

$$I_k = \sum_s \sum_{k'k''} \langle k'|k\rangle \langle k| \langle \psi^s_{k'}|(T^s_{k'})^* T^s_{k''}|\psi_{k''}\rangle |k''\rangle = \sum_s \sum_{k'k''} |T^s_k|^2 \langle \psi^s_{k'}|\psi^s_{k''}\rangle \delta(k,k')\delta(k,k'') = \sum_s |T^s_k|^2, \tag{S31}$$

which illustrates the distribution of E_k. By scanning the state $|k\rangle$, the energy band spectrum can be directly demonstrated.

VI. PHOTON DISTRIBUTIONS AFTER THE MODULATION VIA SLM

The photon distributions after modulated by SLM of different $(k_{\rm exp}, N)$ are shown in Fig. S3. The number of the "petals" of the interfered patterns is $2N$, while the patterns rotate with $k_{\rm exp}$.

VII. DIRECT MEASUREMENT OF THE TOPOLOGICAL WINDING

Under the measurement basis $|k\rangle\langle k| \otimes (\sigma_x, \sigma_y, \sigma_z)$, the output result gives,

$$(I^x_k, I^y_k, I^z_k) = \sum_{k'k''} \langle k'|k\rangle \langle k| \langle \psi^s_{k'}|(T^s_{k'})^* (\sigma_x, \sigma_y, \sigma_z) T^s_{k''}|\psi^s_{k''}\rangle |k''\rangle = \sum_s s(n_x, n_y, n_z)|T^s_k|^2, \tag{S32}$$

where

$$\begin{aligned}
\sigma_x &= |H\rangle\langle H| - |V\rangle\langle V|, \\
\sigma_y &= |A\rangle\langle A| - |D\rangle\langle D|, \\
\sigma_z &= |\circlearrowright\rangle\langle\circlearrowright| - |\circlearrowleft\rangle\langle\circlearrowleft|,
\end{aligned} \tag{S33}$$

and

$$\begin{aligned}
|H\rangle &= \frac{|\circlearrowright\rangle + |\circlearrowleft\rangle}{\sqrt{2}}, \\
|V\rangle &= \frac{|\circlearrowright\rangle - |\circlearrowleft\rangle}{\sqrt{2}}, \\
|A\rangle &= \frac{|\circlearrowright\rangle - i|\circlearrowleft\rangle}{\sqrt{2}}, \\
|D\rangle &= \frac{|\circlearrowright\rangle + i|\circlearrowleft\rangle}{\sqrt{2}}.
\end{aligned} \tag{S34}$$

The topological windings can be revealed by the variations of transmitted peaks.

VIII. EDGE EFFECT AND DISORDER EFFECT

Edge states are topologically protected, an outstanding feature of topological physics. Though the edge effect is weak in our current experiment, the edge states can be investigated in our platform by engineering the operation on different optical modes. For instance, a QWP with a ping hole on its center, as shown in Fig. S4a, can be used to realize such an operation. The radius of the ping hole is set to be 80 μm, which is the same as the waist radius of the Gaussian mode. Only the Gaussian mode with $m = 0$ can pass the hole, and there is no coupling between different circular polarization. While for other modes passing the QWP, the coupling between different circular polarization occurs. The corresponding lattice has an edge at $m = 0$, as shown in Fig. S4b. The system breaks the symmetry at the lattice centre and it can then be viewed as a semi-infinite lattice, where the interface between the non-trivial topological bulk and "vacuum" can support edge states. The numerical DOS without disorder is shown in Fig.S4c, in which the edge states can be clearly seen at $E_k = 0$ or $\pm\pi$.

The disorder is further introduced from the imperfect degeneracy of the cavity, which is given by a random phase $e^{i\Delta\theta_m}$ ($|\Delta\theta_m| < 0.1\pi$) on each optical mode. Such kind of disorder corresponds to a distribution of energies around the main energy and makes the edge energy move to bulk bands. The simulated result is shown in Fig. S4d. The edge state will merge into the bulk state with increasing the disorder strength.

---

† jsxu@ustc.edu.cn

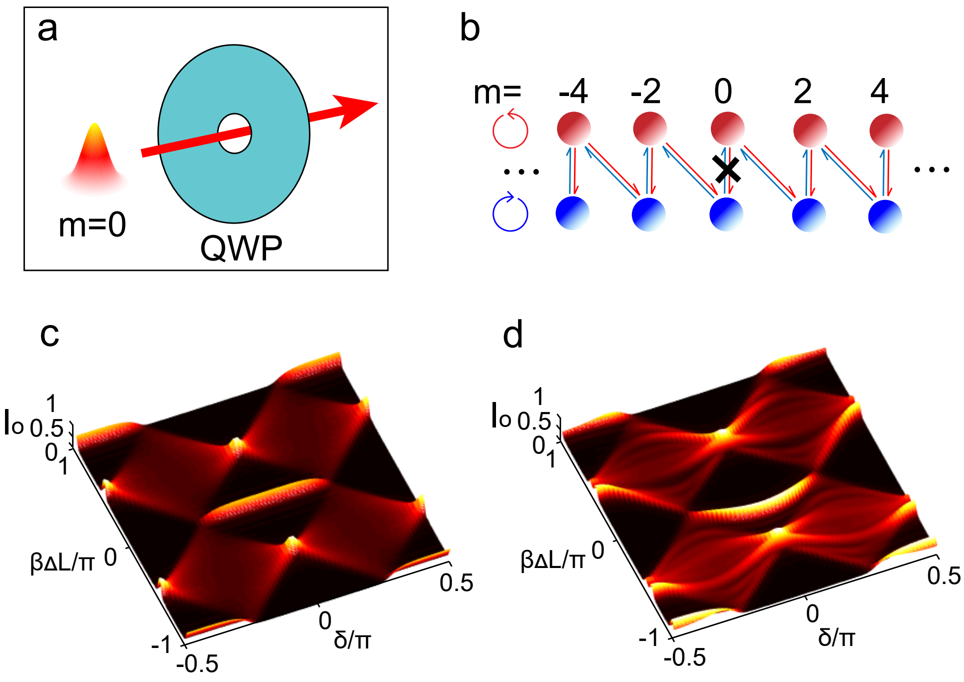

Figure S4. **The density of state (DOS) with edge effects.** **a**. A quarter wave plate (QWP) with a ping hole on the center is placed in the cavity. Only the Gaussian mode with $m = 0$ can pass the hole and there is not coupling between different circular polarization. While for other modes passing the QWP, the coupling between different circular polarization occurs. **b**. The corresponding lattice with an edge at $m = 0$. The lattice breaks into two parts. **c**.The numerical DOS without disorder. **d**.The numerical DOS with disorder.

‡ smhan@ustc.edu.cn

§ cfli@ustc.edu.cn

[1] Arnaud, J.A. Degenerate optical cavities. *Appl. Opt.* **8**, 189-196 (1969)

[2] Arnaud, J.A. Degenerate optical cavities. II: Effect of misalignments. *Appl. Opt.* **8**, 1909-1917 (1969)

[3] Allen, L., Padgett, M. J. The Poynting vector in Laguerre-Gaussian laser modes *Opt. Commun.* **34**, 977 (1995)

[4] Poon, J.K., Scheuer, J., Xu, Y. & Yariv, A. Designing coupled-resonator optical waveguide delay lines. *JOSA B* **21**, 1665-1673 (2004)

[5] Chremmos, I., Uzunoglu, N. Modes of the infinite square lattice of coupled microring resonators. *JOSA A* **25**, 3043-3050 (2008)
